# Surgical versus medical castration in the Bahamas: a male macho paradox

**DOI:** 10.1186/1750-9378-4-S1-S4

**Published:** 2009-02-10

**Authors:** Robin Roberts

**Affiliations:** 1 Department of Surgery, Princess Margaret Hospital, The University of the West Indies School of Clinical Medicine and Research, (The Bahamas), Nassau, Bahamas

## Background

The high prevalence and incidence of prostate cancer is a global phenomenon [1,2]. In the pre-Prostate Specific Antigen (PSA) era, the clinical hallmarks of prostate cancer were late disease presentations and high mortality rates [3]. The documentation of even more advanced presentations and higher mortality rates in males of African ancestry is of great concern for countries like the Bahamas with significant populations of this ethnicity [4-10].

Over the past 15 years, there has been has been a notable change in the clinical presentation of prostate cancer, with more organ confined disease [11,12]. Studies suggest that this documented and consistent trend in the developed countries [13-17], of early disease detection and down stage migration with associated decreasing mortalities, reflect the merits of aggressive PSA screening programs.

In the Bahamas, a country where 85% of the population are of African ancestry, prostate cancer represents both the highest incidences of male malignancy occurrences and cancer specific deaths. Unfortunately, despite the increased campaigns for early detection since the introduction of PSA testing, there has been no down stage migration of clinical presentations of this malignancy in the Bahamas, as has occurred in the developed countries [18]. The cultural ethos of the Caribbean male of African ancestry suggests that health preventative initiatives, inclusive of the digital rectal examination are counter to our valued macho-male image. Men therefore do not avail themselves of the publically accessible early prostate cancer detection programs.

With this high incidence of advance disease and noting that hormonal therapy remains the first treatment of choice, we sought to determine the most common treatment modality employed in our institution with regards to surgical versus medical castration. Emphasizing the need for cost effective and affordable care in our developing country, would men of African ancestry in a macho dominated society opt to have surgical castration as the preferred treatment?

## Methods

All men presenting with advanced prostate cancer at the government-owned public health facility, the Princess Margaret Hospital, are informed by the Consultant Urological Surgeon of the various medical and surgical hormonal options and their advantages and disadvantages. They are informed also that the institution would provide the surgical option of bilateral orchiectomies at no charge, but the cost of the medical treatment option must be borne by the patient.

At the only two hospitals on New Providence Island in the Bahamas, the Princess Margaret Hospital, (450 beds) and the privately-owned Doctors Hospital (70 beds), all pathology reports for biopsy proven cancers and the operative log for the number of surgical castration procedures were reviewed during a thirteen years period from 1987 to 2000. The data base is compiled from that of a solo urology service providing care in both the private and public sectors in the Bahamas; this service represents 70% of the urological health care delivered in the country. It is important to note that almost 70% of the population of the Bahamas resides on New Providence Island on which the capital city of the Bahamas is located.

## Results

There were 535 pathology-diagnosed cases of prostate cancer identified. 275 bilateral orchiectomies were performed in patients presenting with advance prostate cancer during this period, an average of 21.5 bilateral orchiectomies performed annually.

For the five years period 2003 to 2007 at the government's public hospital, all cases of pathology proven prostate cancer were reviewed. There were 363 documented cases of prostate cancer. During this period, there were 103 cases of bilateral orchiectomies recorded in the operative log of the hospital, averaging 20.6 cases per year. The frequency of bilateral orchiectomies performed annually was similar to that of the thirteen year period.

This high rate of hormonal treatment is an indication of the continuing trends of advance disease as the initial presentation of males diagnosed with prostate cancer in the Bahamas. The trend of increasing annual mortality rates for prostate cancer has continued unabated for the past 15 years, contrary to that of the developed countries; this is well documented in the annual cancer mortality reports by the Health Information and Research Unit of the Ministry of Health and Social Services of the Bahamas.

## Conclusion

This study concludes that men in the Bahamas with advanced prostate cancer would opt for surgical castration when presented 'positively' as the preferred treatment. These findings are contrary to the perception of the macho-male image of the Caribbean male and invite further studies into the complex psyche of our Bahamian males.

## Competing interests

The author declares that they have no competing interests.
